# Hospitalized cancer patients with acquired long QT syndrome-a matched case-control study

**DOI:** 10.1186/s40959-020-0057-2

**Published:** 2020-02-14

**Authors:** Yajuan Lin, Haixu Yu, Fei Liu, Cheng Chen, Yanli Zhang, Binhao Wang, Yiheng Yang, Ying Liu, Li Zhang, Yunlong Xia

**Affiliations:** 1 Department of Cardiology, First Affiliated Hospital of Dalian Medical University, Zhongshan Road 222, PO Box 116011, Dalian, Liaoning China; 2 Department of Cardiology and Institute of Vascular Medicine, Peking University Third Hospital, Beijing, China; 3 Arrhythmia Center, Ningbo First Hospital, Ningbo, Zhejiang China; 4 Sidney Kimmel Medical College of Thomas Jefferson University, Philadelphia, PA USA; 5 Lankenau Institute for Medical Research, 100 Lancaster Avenue, Wynnewood, PA 19096 USA

**Keywords:** Acquired long QT syndrome, Cancer, Cardiotoxicity, All-cause mortality

## Abstract

**Background:**

Our recent study has revealed that many hospitalized patients with acquired long QT syndrome (ALQTS) are cancer patients. This study aims to determine the risk factors and outcomes of hospitalized cancer patients with ALQTS.

**Methods:**

We performed a matched case-control study within a cohort of 10,180 cancer patients hospitalized between September 2013 and April 2016. Among them, 150 patients defined as having severe ALQTS with a markedly prolonged QT interval (QTc ≥ 500 ms) were compared with 293 age-, sex- and cancer-type-matched controls (non-ALQTS). Death as the endpoint was followed for up to 2 years. Cox regression and Kaplan-Meier survival analyses were performed to assess the effects of particular clinical variables on all-cause mortality. Multivariate logistic regression was performed to calculate odds ratios (OR) for various predictors of QT prolongation.

**Results:**

The mortality was significantly higher in ALQTS group (63.3% vs. 33.4%). Hypertension, hypokalemia, hypocalcemia, QT-prolonging drugs, infection, anemia, anti-microtubule agents were contributing factors to ALQTS. Renal insufficiency, male gender and hypokalemia were found to be independent risk factors for all-cause mortality in ALQTS group.

**Conclusion:**

Markedly prolonged QT interval was seen in 1.5% of hospitalized cancer patients. The all-cause mortality was high in cancer patients with severe ALQTS.

## What’s new

Cardiotoxicity related to cancer therapy is of rising concern since it negatively impacts the prognosis. The 2016 European Society of Cardiology guideline described updated standard therapies for acquired long QT syndrome (ALQTS) associated with cancer therapy. However, evidence-based data regarding ALQTS in hospitalized cancer patients is limited.

## Clinical implications

In our study cohort, markedly prolonged QT interval was seen in 1.5% of hospitalized cancer patients. The all-cause mortality was high in cancer patients with severe ALQTS.

## Introduction

With advances in early detection and therapeutics, the cancer survival rate continues to rise worldwide. Cancer survivors, nevertheless, are susceptible to cardiovascular diseases and cardiotoxicity of cancer drugs [1, 2]. Cardiovascular disorder is a major cause of morbidity and mortality in cancer survivors [3]. These patients are prone to various types of arrhythmias, including tachycardias, bradycardias and conduction disorders [4, 5]. Sometimes cardiac arrhythmias can be life-threatening [6]. In this regard, QT prolongation caused by acquired factors associated with cancer therapeutics, termed Acquired Long QT Syndrome (ALQTS), is a key risk state that has been long overlooked in both cardiology and oncology. Using the Pro-QTc score in an institution-wide QT alert system could help identify the high-risk of mortality with markedly prolonged QT interval (QTc ≥ 500 ms). In 2017, our group reported cancer to be one of the major factors associated with all-cause mortality in ALQTS [7]. Thus, this study aimed to investigate the impact of ALQTS on the prognosis in hospitalized cancer patients. A priori, we hypothesized that the outcome of cancer patients with ALQTS would be poor, affected by various clinical factors.

## Materials and methods

### Study population

In this matched case-control study, we retrospectively enrolled hospitalized cancer patients with severe ALQTS from September 2013 to April 2016 in the First Affiliated Hospital of Dalian Medical University. Study population was separated into ALQTS group and control group. The inclusion criteria for the ALQTS group were: Hospitalized cancer patients (age > 18 years) from Hematology-Oncology or other departments showing QTc (Bazett’s) ≥ 500 ms without a family history of inherited LQTS, unexplained syncope, cardiac arrest, or sudden death. The exclusion criteria included QRS duration > 120 ms, presence of complete left or right bundle branch blocks, intraventricular conduction delays or ventricular pacing. Moreover, ECG showing atrial fibrillation/flutter, second-degree and complete atrial-ventricular blocks, severely sinus, atrial and ventricular tachyarrhythmia, and acute coronary syndrome with dynamic ST-T that interfered accurate QT assessment were excluded. The inclusion criteria for the control group were same as the ALQTS group, except all had a normal QT interval (350 ms < QTc ≤ 440 ms) during hospitalization. The age, sex and the cancer diagnosis were comparable between ALQTS and control groups. This study was approved by the Ethics Committee of the First Affiliated Hospital of Dalian Medical University.

### ECG evaluation

According to the inclusion and exclusion criteria, we retrieved electronic records of standard resting 12-lead ECG parameters from the Muse System (7.1.1 edition), including heart rate (HR) and QTc. ECG were recorded with a paper speed of 25 mm/s and voltage of 10 mm/1 mV in the supine position, using GE Healthcare MAC 5500 ECG diagnosis systems (India). If differences in the QTc interval exceeded 20 ms between the machine and hand measurement, the interval was corrected manually, especially in cases with complex T wave morphologies. In these cases, QT intervals were measured by an experienced QT investigator from lead II, V5 or the lead with the longest QT interval. If several ECG recordings during hospitalization were available, the longest QTc was selected.

### Clinical evaluation

From the electronic medical records, we retrieved data including cancer diagnosis, classification and staging, medical history, chemotherapy drugs and presence of QT-prolonging factors such as electrolyte disorders and use of QT-prolonging drugs. Laboratory tests taken within 48 h before or after ECG recording were evaluated, including serum potassium (normal range 3.5–5.3 mmol/L) and serum calcium (2.02–2.6 mmol/L). QT-prolonging drugs used within 7 days of the ECG evaluated in this study were screened through the website (https://www.crediblemeds.org).

### Follow-up

In this study, all-cause death was determined as research endpoint. Telephone survey was conducted after permission obtained from study participants or their legally authorized representatives. When death had occurred, the possible causes were investigated.

### Application of the pro-QTc score system

We employed the Pro-QTc Score to explore the risk of death in patients with QTc ≥ 500 ms. QT-prolonging diagnoses and conditions include cardiomyopathy, acute coronary syndrome, prolonged QT interval, congestive heart failure, bradycardia, diabetes mellitus, stroke, hypokalemia, hypocalcemia, hypomagnesemia, female gender, old age, and several QT-prolonging drugs, which have been compiled in the QT drug list by the Arizona Center for Education and Research on Therapeutics (AZCERT). Each measure in the score system was scaled as 1 point [8]. Pro-QTc scores ≥4 were considered predictive of high mortality risk.

### Statistical analyses

Continuous data were expressed as mean ± SD, and comparative analysis was performed by Student’s *t*-tests for independent samples. The Mann-Whitney *U* Test was applied when variables were non-normal distributions. Categorical variables were expressed as absolute (n) and relative proportions (%), and were analyzed using the *χ*^2^ (Chi-square) test. 2-tailed statistical significance was considered when *p* <  0.05. Multivariate logistic regression was performed to calculate odds ratios (OR) for various predictors of QT prolongation. For this procedure, variables were selected on the following based on clinical relevance and presence of *p* values < 0.1 in univariate analysis. The univariate cumulative probability of all-cause mortality during hospital stay and up to 2-year follow-up was assessed by the Kaplan-Meier survival curve using Log-rank statistics. The Cox proportional hazard survival model was used to evaluate the effect of clinical factors on the endpoint. Statistical analyses were performed using SPSS software (version 22.0, SPSS Inc., Chicago, IL).

## Results

### Incidence of severe ALQTS in hospitalized cancer patients and their clinical characteristics

Between September 2013 and April 2016, we identified 200 subjects with ALQTS from a total of 10,180 cancer patients hospitalized in the hematology-oncology departments. After applied exclusion criteria and manually checking QT measurement performed by experienced QT investigators, 150 ALQTS patients remained in the study. Compared with age, sex, and cancer type matched 293 non-ALQTS patients, the incidence of hospitalized cancer patients with severe ALQTS was 1.5% (150/10180).

As to the cancer diagnosis in this study cohort, hematological cancer accounted for 25%, lung cancer 21%, gastrointestinal cancer 20%, breast cancer 17%, gynecologic cancer 7%, urological cancer 3%, prostate cancer 1%, and other cancers 5%. The baseline characteristics of the ALQTS and non-ALQTS groups was summarized in Table 1.

**Table 1:** Clinical characteristics in hospitalized malignant cancer patients with and without severe ALQTS

ECG and clinical aspects	ALQTS (*n* = 150)	Non-ALQTS (*n* = 293)	*p* value
Age (years)	62 ± 13	62 ± 13	0.331
Gender			
Female	58%	58%	0.887
Male	42%	42%	
Comorbidities			
Hypertension	29.3%	16.4%	0.001
Heart Failure	6.7%	0.7%	< 0.001
Arrhythmia	6.7%	2.7%	0.047
Renal Insufficiency	5.3%	2.7%	0.165
Infection	21.3%	5.1%	< 0.001
Type 2 mellitus	16.0%	9.6%	0.046
Neurologic diseases	10.7%	3.4%	0.002
Anemia	12.0%	1.4%	< 0.001
ECG Data			
Heart rate (bpm)	88 ± 17	72 ± 12	< 0.001
QTc (ms) φ	520 (20)	422 (18)	< 0.001
Laboratory Test			
Hypokalemia	31.7%	5.2%	< 0.001
Hypocalcemia	22.7%	4.9%	< 0.001
Medications			
QT-prolonging drugs	31.3%	6.8%	< 0.001
Chemotherapeutic drugs			
Anti-microtubule agents	36%	28%	0.084
Platinum	34%	31.1%	0.530
Antimetabolites	33.3%	27.3%	0.187
Alkylating agents	29.1%	17.7%	0.215
Anthracyclines	28%	28.1%	0.151
Arsenic trioxide	2%	1.4%	0.612

Values are expressed as mean ± SD or median (interquartile range) unless otherwise indicated. φ Median (inter-quartile range) is shown. *Abbreviations*: *ALQTS* acquired long QT syndrome, *ECG* electrocardiograph

### Outcomes and risk factors for QT-prolongation in hospitalized cancer patients with severe ALQTS

**Fig. 1:**
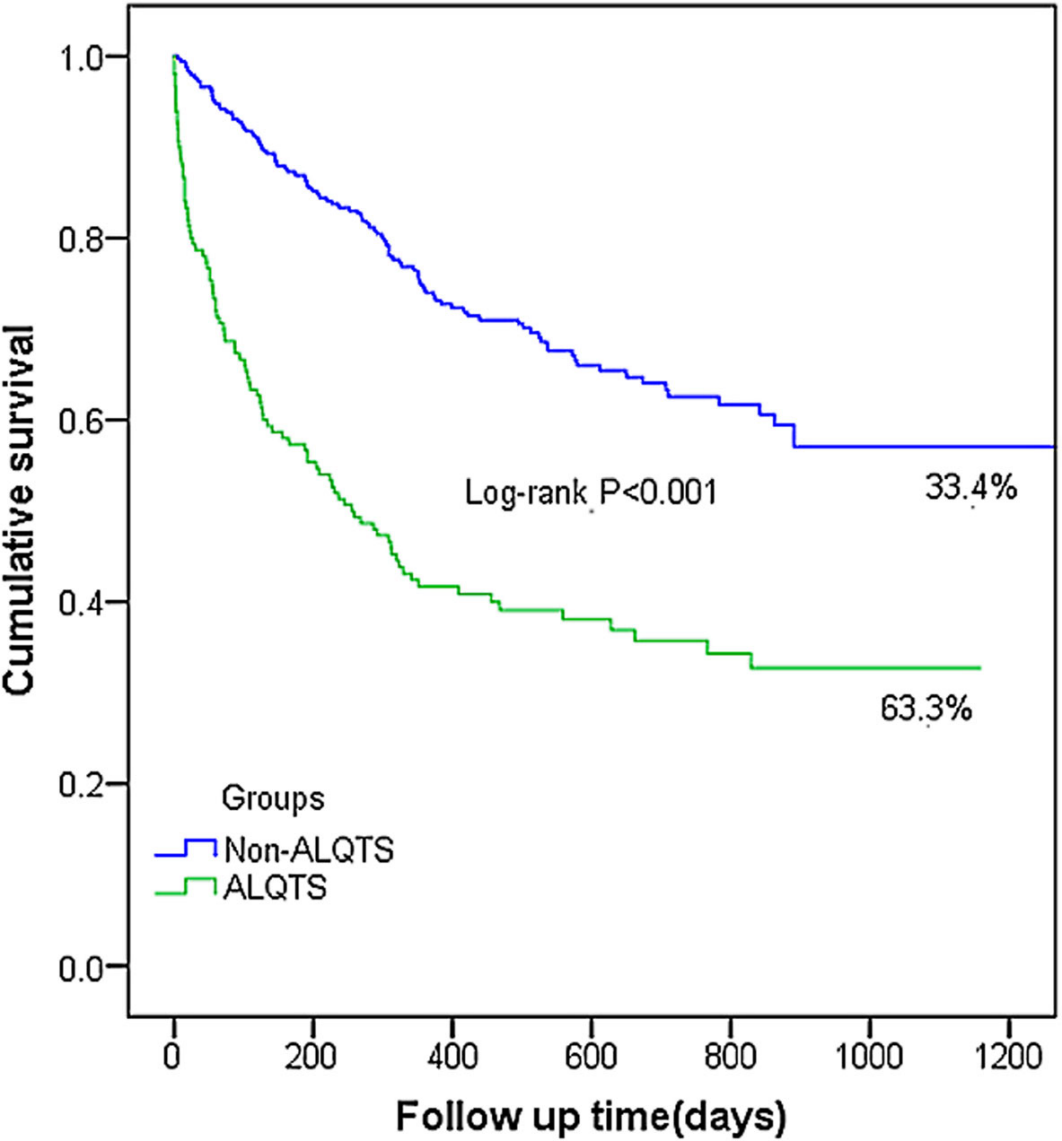
Kaplan-Meier Survival Curve in Hospitalized Patients with and without Severe ALQTS. Kaplan-Meier analysis shows that cancer patients with severe ALQTS had much higher all-cause mortality. Abbreviations: ALQTS, acquired long QT syndrome

Kaplan-Meier analysis shows that cancer patients with severe ALQTS had much higher all-cause mortality (Fig. 1) compared to age-, sex- and cancer diagnosis-matched hospitalized cancer patients with normal QT intervals (63.3% vs. 33.4%, *p* <  0.001). Clinical factors associated with QT prolongation in our study cohort are shown in Table 2.

**Table 2:** Clinical factors associated with QT prolongation in hospitalized cancer patients*

Variables	OR (95%CI)	*p* value
Hypertension	2.433 (1.319–4.489)	0.004
Infection	3.456 (1.471–8.121)	0.004
Anemia	4.879 (1.325–17.961)	0.017
Hypokalemia	6.271 (3.007–13.080)	< 0.001
Hypocalcemia	2.360 (1.028–5.445)	0.043
QT-prolonging drugs	5.083 (2.544–10.155)	< 0.001
Anti-microtubule agents	2.857 (1.658–4.925)	< 0.001

*Using multiple logistic regression analysis. *Abbreviations*: *OR* odds ratio, *CI* confidence interval

### QT-prolongation and chemotherapeutic drugs

As shown in Tables 1, 31.3% of patients in the ALQTS group used QT-prolonging drugs, including diphenhydramine (26%), famotidine (23%), quinolone antibiotics (21%), and furosemide (19%). Additionally, there were no significant differences among chemotherapeutic drugs between the ALQTS and non-ALQTS groups.

### Clinical characteristics and all-cause mortality contributors between non-survivors and survivors in hospitalized cancer patients with severe ALQTS

Comparisons between survivors and non-survivors in the ALQTS group are presented in Table 3. Multivariable Cox regression was performed between non-survivors and survivors in hospitalized cancer patients with severe ALQTS (Table 4). The Cox proportional hazard survival model revealed that male (HR 1.643, 95%CI 1.023–2.640, *p* = 0.040), renal insufficiency (HR 5.358, 95%CI 1.984–14.473, *p* = 0.001), and hypokalemia (HR 2.315, 95%CI 1.417–3.781, *p* = 0.001) were major contributors to all-cause mortality.

**Table 3:** Clinical characteristics in hospitalized cancer patients with severe ALQTS between Non-survivors and survivors

Parameters	Non-survivors (*n* = 95)	Survivors (*n* = 55)	*P* Value
Age, years	66 ± 10	56 ± 14	0.000
≥ 70 years	36.8%	18.2%	0.016
Gender			
Female	49.5%	72.7%	0.005
Male	50.5%	27.3%	
HR, bpm	90 ± 19	85 ± 12	0.062
QTc, ms	513	509	0.243
Hypokalemia	40.4%	17%	0.004
Hypocalcaemia	26.1%	17%	0.209
Use of QT-prolonging drugs	36.4%	28.4%	0.312
Renal insufficiency	8.4%	0.0%	0.027
Infection	26.3%	12.7%	0.05
Anemia	15.8%	5.5%	0.061
Arrhythmia	5.3%	9.1%	0.365
Heart Failure	7.4%	5.5%	0.651
Hypertension	29.5%	29.1%	0.96

*Abbreviations*: *ALQTS* acquired long QT syndrome, *HR* heart rate

**Table 4:** Cox proportional hazard survival between non-survivors and survivors in the severe ALQTS group

	Univariate analysis		Multivariate analysis	
	HR (95%CI)	*P* value	HR (95%CI)	*P* value
Male	1.793(1.198–2.682)	0.005	1.643(1.023–2.640)	0.040
Age ≥ 70 years	1.595 (1.051–2.420)	0.028	1.034 (0.624–1.715)	0.896
Pro-QTc ≥ 4 score	1.839 (1.155–2.929)	0.010	1.090 (0.561–2.116)	0.799
Hypokalemia	2.403 (1.565–3.689)	< 0.001	2.315 (1.417–3.781)	0.001
Hypocalcemia	1.833 (1.136–2.959)	0.013	1.258 (0.697–2.272)	0.446
Renal failure	4.405 (2.097–9.252)	< 0.001	5.358 (1.984–14.473)	0.001
Infection	1.970 (1.243–3.123)	0.004	1.423 (0.854–2.373)	0.176
Anemia	2.388 (1.367–4.169)	0.002	1.717 (0.837–3.523)	0.140

*Abbreviations*: *ALQTS* acquired long QT syndrome, *HR* Hazard Ratio, *CI* confidence interval

### Application of the pro-QTc score

**Fig. 2:**
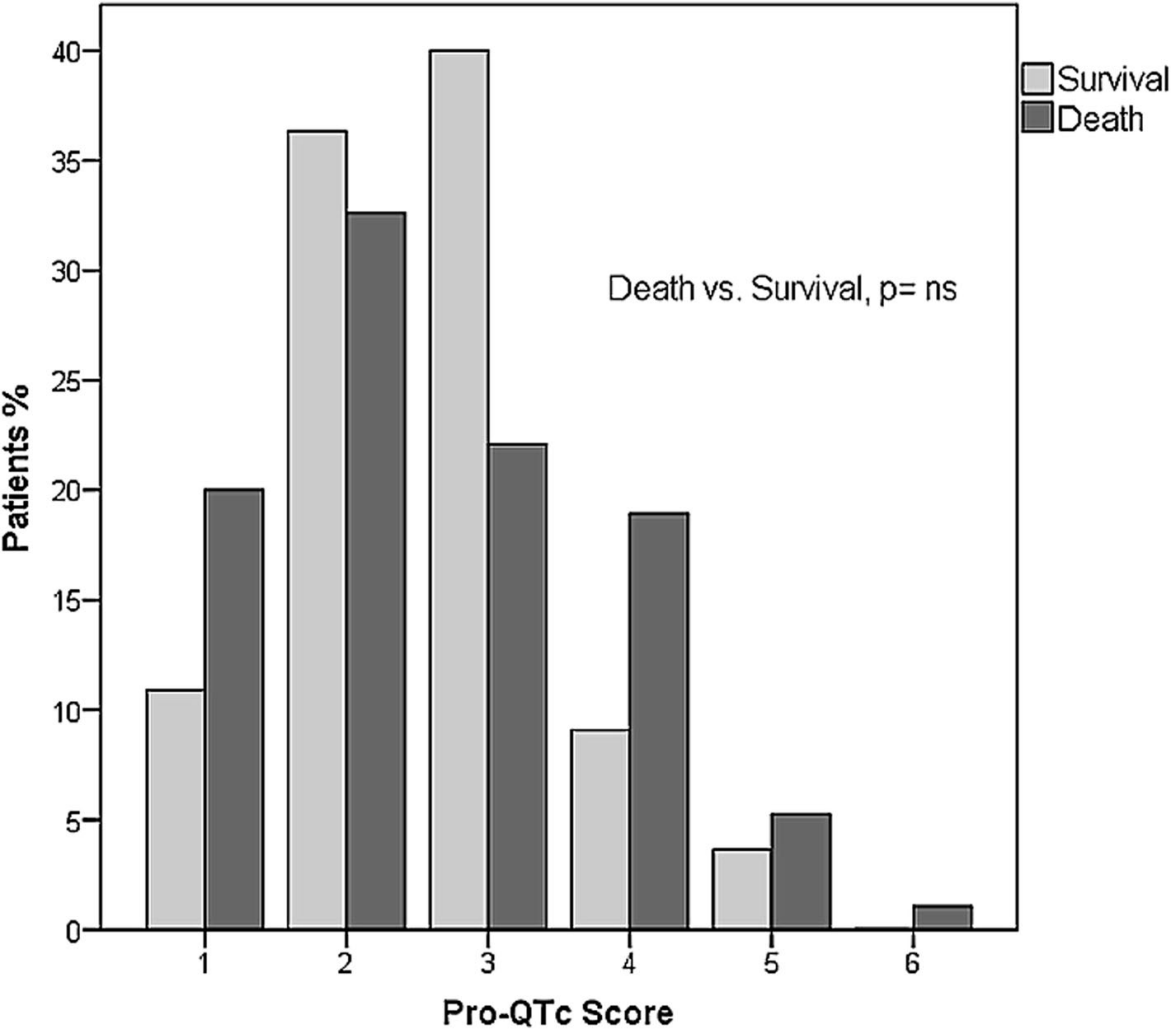
Pro-QTc score in patients with severe ALQTS. There were no significant/statistical difference between two groups. Abbreviations: ALQTS, acquired long QT syndrome

In our study, we applied the Pro-QTc score, proposed by the Mayo Clinic. Figure 2 shows the Pro-QTc scores of patients with severe ALQTS. A significant difference was found between non-survivors and survivors in the group with severe ALQTS group in univariate Cox hazard survival model; however, no difference was ascertained after multivariate adjustment (Table 4).

## Discussion

In this study, we found that severe ALQTS (QTc ≥ 500 ms) accounts for 1.5% of cancer patients for in-hospital treatment. Among cancer patients, the all-cause mortality is significantly higher in those with ALQTS than those without QTc prolongation. Generally, ALQTS subjects are more severe than those with normal QTc interval (Table 1), with higher frequency of electrolyte imbalances, more use of QT-prolonging drugs and more patients suffering from cancer-related infections, anemia, and concomitant diseases.

Severe ALQTS in hospitalized patients is seen 0.7% as we reported previously [7]. The prevalence of severe ALQTS in cancer patients revealed in this study is 2-fold (1.5%) higher indicating delayed ventricular repolarization is much more profound among cancer patients. This finding, however, does not necessarily mean that cancer is a direct cause of QT prolongation. The higher prevalence of ALQTS in cancer patients is likely contributed by 1) presence of QT-prolonging electrolyte imbalance such as hypokalemia and hypocalcaemia (Table 1); 2) use of QT-prolonging drugs; 3) baseline conditions such as hypertension. Most of cancer patients were elders and many had a history of hypertension. Left ventricular hypertrophy resulted from hypertension is a major cause of ALQTS [9, 10]. Chemotherapy can impair immune system. Thus, post-chemotherapy patients are prone to infections. Infection with inflammatory environment can prolong QT interval due to the effect of inflammatory cytokines (interleukin-6, tumor necrosis factor-α, interleukin-1) on potassium and calcium channels [11–14]. QT-prolonging antibiotics such as quinolone antibiotics are commonly prescribed in cancer patients. Anemia is very common in cancers patients especially in late stage. The decreased hemoglobin level can cause tachycardia-cardiomyopathy and increased myocardium mass is associated with QT prolongation [15–17].

As demonstrated in Table 1 cancer patients with severe ALQTS are generally sicker than those without ALQTS. Thus, it is in no surprise that they had a poor outcome measured by a significantly higher all-cause mortality (Fig. 1). In this study, we found that renal insufficiency is one of the contributors to all-cause mortality in cancer patients with ALQTS. Hypokalemia is common in patients with renal insufficiency immediately post dialysis and in patients using potassium-loss diuretics. Therefore, on the basis of previous and our study, we speculate that eliminating the use of QT-prolonging medications and monitoring modifiable QT-prolonging risk factors such as management of electrolyte disturbance are beneficial for prognosis especially in cancer patients with severe ALQTS.

The application of chemotherapeutic drugs is indispensible and complex in cancer patients. Anthracyclines (doxorubicin) [18], arsenic trioxide [19] vandetanib [6, 20] and molecular-targeted drugs [21–24] have been confirmed to prolong the QTc interval. As shown in Table 1, our study showed that, except for anti-microtubule agents which has been reported to be linked to QTc prolongation [25, 26], the rest of chemotherapeutic QT-prolonging drugs are not statistically significant in our study. The insignificance may be related to the small number of patients in terms of each cancer drug used.

In this study, Pro-QTc score ≥ 4 [7] failed to predict the all-cause mortality in cancer patients. It is perhaps that Pro-QTc was designed to pick up risk factors of sudden arrhythmic death. Most of our non-survival cancer patients died of cancer itself. Prolonged QT interval may reflect the disease severity rather than the direct cause of death in most of cancer non-survivors. Nevertheless, most of QT-prolonging factors are modifiable. Further study is warranted to determine whether eliminating certain risk factors exerts influence on patient outcomes. Establishing an accurate evaluation model to predict mortality risk in cancer patients with ALQTS is urgent.

### Limitation

Our findings should be interpreted in the context of the following limitation. Study outcomes were defined as all-cause mortality in this retrospective study, with limited electrophysiological data after patients discharged from hospital. Because of the use of telephone follow-up, specific causes of death cannot be obtained and data analysis related to cardiovascular death is lacking. Further studies are required to examine the effect of ALQTS on the sudden death in hospitalized cancer patients.

## Conclusions

Markedly prolonged QTc interval was seen in 1.5% of hospitalized cancer patients. The all-cause mortality was high in cancer patients with severe ALQTS.

## Data Availability

The datasets used and/or analyses during the current study are available from the corresponding author on reasonable request.

## Abbreviations

ALQTS: Acquired long QT syndrome
CI: Confidence interval
HR: Heart rate/Hazard ratio
OR: Odds ratio
